# MRI Study of Minor Physical Anomaly in Childhood Autism Implicates Aberrant Neurodevelopment in Infancy

**DOI:** 10.1371/journal.pone.0020246

**Published:** 2011-06-08

**Authors:** Charlton Cheung, Grainne M. McAlonan, Yee Y. Fung, Germaine Fung, Kevin K. Yu, Kin-Shing Tai, Pak C. Sham, Siew E. Chua

**Affiliations:** 1 Department of Psychiatry, The University of Hong Kong, Pokfulam, Hong Kong; 2 Department of Radiology, The University of Hong Kong, Pokfulam, Hong Kong; 3 State Key Laboratory for Cognitive Neuroscience, The University of Hong Kong, Hong Kong; 4 Harvard School of Dental Medicine, Harvard University, Boston, Massachusetts, United States of America; University of Regensburg, Germany

## Abstract

**Background:**

MPAs (minor physical anomalies) frequently occur in neurodevelopmental disorders because both face and brain are derived from neuroectoderm in the first trimester. Conventionally, MPAs are measured by evaluation of external appearance. Using MRI can help overcome inherent observer bias, facilitate multi-centre data acquisition, and explore how MPAs relate to brain dysmorphology in the same individual. Optical MPAs exhibit a tightly synchronized trajectory through fetal, postnatal and adult life. As head size enlarges with age, inter-orbital distance increases, and is mostly completed before age 3 years. We hypothesized that optical MPAs might afford a retrospective ‘window’ to early neurodevelopment; specifically, inter-orbital distance increase may represent a biomarker for early brain dysmaturation in autism.

**Methods:**

We recruited 91 children aged 7–16; 36 with an autism spectrum disorder and 55 age- and gender-matched typically developing controls. All children had normal IQ. Inter-orbital distance was measured on T1-weighted MRI scans. This value was entered into a voxel-by-voxel linear regression analysis with grey matter segmented from a bimodal MRI data-set. Age and total brain tissue volume were entered as covariates.

**Results:**

Intra-class coefficient for measurement of the inter-orbital distance was 0.95. Inter-orbital distance was significantly increased in the autism group (p = 0.03, 2-tailed). The autism group showed a significant relationship between inter-orbital distance grey matter volume of bilateral amygdalae extending to the unci and inferior temporal poles.

**Conclusions:**

Greater inter-orbital distance in the autism group compared with healthy controls is consistent with infant head size expansion in autism. Inter-orbital distance positively correlated with volume of medial temporal lobe structures, suggesting a link to “social brain” dysmorphology in the autism group. We suggest these data support the role of optical MPAs as a “fossil record” of early aberrant neurodevelopment, and potential biomarker for brain dysmaturation in autism.

## Introduction

Minor physical anomalies (MPAs) occur more frequently in neurodevelopmental disorders such as autism [1], [2], [3]. This is believed to be because the skin and brain originate from the same neuroectodermal layer that is destined to become brain tissue. These events occur during the very same time window i.e. in the first trimester of fetal life. Since “cranio-facial” development is synchronized in early life, this means that a convenient biomarker might exist to ‘mirror’ aberrant neurodevelopment.

Minor physical anomalies (MPAs) are neurodevelopmental markers which manifest as unusual morphological features of the face or physique [4]. They occur in more than 14% of healthy newborn [5], [6] but can be as high as 60% in neurodevelopmental disorders such as schizophrenia, autism, hyperactivity, epilepsy, or mental retardation [1], [7]. MPAs have their origin in the first trimester, established by around week 16 [8] or up to week 22 of embryonic life. Minor physical anomalies are stable over time and can be studied efficiently from early childhood onwards [9]. They are known to be strongly correlated with brain structural abnormalities; developmentally delayed individuals are more than twice as likely (29%) as developmentally normal individuals to have structurally abnormal MRIs (14%) [5]. Thus MPAs they may serve as markers of concomitant disruption of brain development [10]. Consistent with this, a higher incidence of MPAs such as hypertelorism in autism (increased inter-orbital distance, or more readily measured inter-pupillary or inter-canthal distances) was first reported in 1977 [2]. A recent study of 24 children with autistic spectrum disorder compared with 24 healthy children has also lent support to the possibility that aberrant intrauterine development leads to MPAs in autism [3].

Traditionally, MPAs have been evaluated by visual examination of the body using the Waldrop scale [11], [12]. However, the Waldrop method has limitations, including low sensitivity, inter-rater reliability and ethnic differences [13]. By convention, MPAs require face-to-face evaluation but this engenders potential subjective bias. Recent work suggests that for an assessment that is blind to diagnosis, magnetic resonance imaging (MRI) can be used; for example MRI can help to quantify binocular diameter in patients with schizophrenia and has been found to be practical, reliable, and valid [14]. One study correlating a total MPA score with brain grey matter volumes in a first episode multi-ethnic psychosis sample reported prefrontal and subcortical grey volume deficits associated with MPAs [15]. MRI has a number of advantages for evaluating cranio-facial MPAs: it is ‘blind’ to ethnicity, ‘blind’ to subject identity, and can directly compare brain morphology with MPAs for each and every subject. Consequently, MRI can perform an integrated evaluation of cranio-facial morphology. So far, only one group has used MRI to measure inter-orbital and inter-pupillary distance in autism and found no significant difference by comparison with healthy controls, except that for individuals with autism and low IQ there was a link with hypotelorism [16]. However, that study fixed inter-orbital distance as 4 slices behind the maximum inter-pupillary distance (though this might be variable across individuals), and did not evaluate the relationship between inter-orbital distance and underlying brain structural indices.

In the current study, we propose that optical MPAs can serve as a putative biomarker to provide an accessible ‘window’ onto early neurodevelopment. The rationale for this is simple but compelling. First, it is known that the causes of autism may be linked *inter alia* to genes acting early in development or prenatal environmental risk factors [17], therefore it follows that a putative biomarker may well have neurodevelopmental origins. Second, MPAs are strongly associated with brain structural abnormalities which are also correlated with clinical severity in autism [5]. Third, fetal head size expansion is linked to enlargement of the neurocranium and antero-posterior cranial base, and all this takes place rapidly in early postnatal life to attain near-adulthood dimensions. Fourth, early head size expansion is mirrored by a contraction of the optic chiasm (ie the angle between the left and right optic nerves) in a time-locked trajectory : in the embryo this inter-optic angle begins at 180 degrees, reduces to 71 degrees at birth to confer binocular vision, and is fixed at 68 degrees by adulthood [18], [19]. Simultaneously, the inter-orbital distance increases to reach 50% of adulthood dimensions by age 3 [18], [20]. Thus it is plausible that optical MPAs provide an accessible ‘window’ onto early neurodevelopment.

In the present study, we describe the use of MRI to carefully measure inter-orbital distance. Considering that MPAs are established in intra-uterine life, that head size expansion is accompanied by an increase in inter-orbital distance increase in infancy, and there is dramatic head size expansion early in autism, we hypothesize that, in older children with autism, this will be reflected in greater inter-orbital distance. In addition, we use voxel-based methods to determine brain regions where grey matter anatomy may have been subject to the same developmental pressure in autism.

## Methods

The total sample comprised 91 children from our database who had trimodality MRI data available for analysis (T1, T2/PD described below). Thirty-six children had an independent clinical diagnosis of autism spectrum disorder (6 females), were aged 7–16 years, and attending mainstream schools. Verbal IQ was estimated on the verbal subset of the WISC. Thirty of these passed cut-off for autism on all three domains of the ADI-R (Autism Diagnostic Interview – Revised version (ADI-R) A, B, C domains © Western Psychological Press, 2003). We also included 6 children classified as having pervasive developmental disorder not otherwise specified on the ADI-R (5 missed cut-off on 1 domain by 1 point). Exclusion criteria included a clinical diagnosis of co-morbid psychiatric or medical condition such as epilepsy, history of head injury, or clinically detectible genetic syndrome associated with autism such as Fragile X syndrome. Fifty-five age- and gender-balanced healthy controls (8 females) from local mainstream schools had been screened for major psychiatric illness using the structured parental interview of the Cantonese Diagnostic Interview Schedule for Children – Fourth version (DISC-IV), [62]. Verbal IQ estimated on the verbal subset of the WISC was available for all but 4 children. All subjects involved in the study provided assent or informed consent for the protocol which was approved by the Hong Kong West Cluster Institutional Review Board.

### MRI acquisition and analysis

All MRI scans were obtained on a GE Signa 1.5 Tesla system (General Electric, Milwaukee, WI, USA) in Queen Mary Hospital, Hong Kong.

T1-weighted scans were used for measurement of MPAs since they give clear grey-white definition of soft tissue and muscle landmarks. The MRI scanner prioritized clinical scan image quality such that in the course of this research study the T1 acquisition sequences were of two types. These were evenly distributed across the patient and control groups so that two thirds of the subjects in each group were scanned with sequence 1 (TR/TE = 9/1.9 ms, 1.5 mm contiguous coronal slices, matrix = 256×256, FOV = 22 cm2, Inversion Time = 450, NEX = 1), and the rest of subjects with sequence 2 (TR/TE = 11.4/4.2 ms, 1.2 mm contiguous axial slices, matrix 256×256, FOV = 24 cm2, Inversion Time = 600, NEX = 1). Although the T1 protocol variation was not expected to affect our results, we took care to enter T1 protocol as a covariate in the analyses of optical parameters.

Interleaved dual-echo fast-spin echo (FSE) PD/T2 scans were used for voxel based morphometry. We have previously described the use of this protocol for whole brain voxel based analysis [24], [25], [63], [64], [65]. All subjects were scanned with the following parameters: repetition time, TR = 3000 ms, echo times, TE1 = 20 ms (proton density weighted images) and TE2 = 100 ms (T2-weighted images). Images were acquired along the AC-PC line, with contiguous slices 0.859 mm in-plane and 3 mm thick.

Scanning time for each subject was approximately 20 minutes. Each scan was screened by a consultant radiologist to exclude clinically significant abnormalities. All MRI scans were then assigned numerical codes to ensure that analyses were performed blind to group status.

### Measurements

T1 images were scrutinized along the sagittal and coronal planes in order to observe for rotational tilt due to ‘roll’ or ‘pitch’ in each plane respectively. For each image, a transformation matrix quantifying the tilt angle was constructed so that realignment could be achieved using SPM2 software (Wellcome Department of Imaging Neuroscience, London (http://www.fil.ion.ucl.ac.uk). A reverse transformation could then be applied to the image to adjust tilt. This re-aligned image was resliced to a new volume with isotropic 1×1×1 mm voxels.

**Figure 1:**
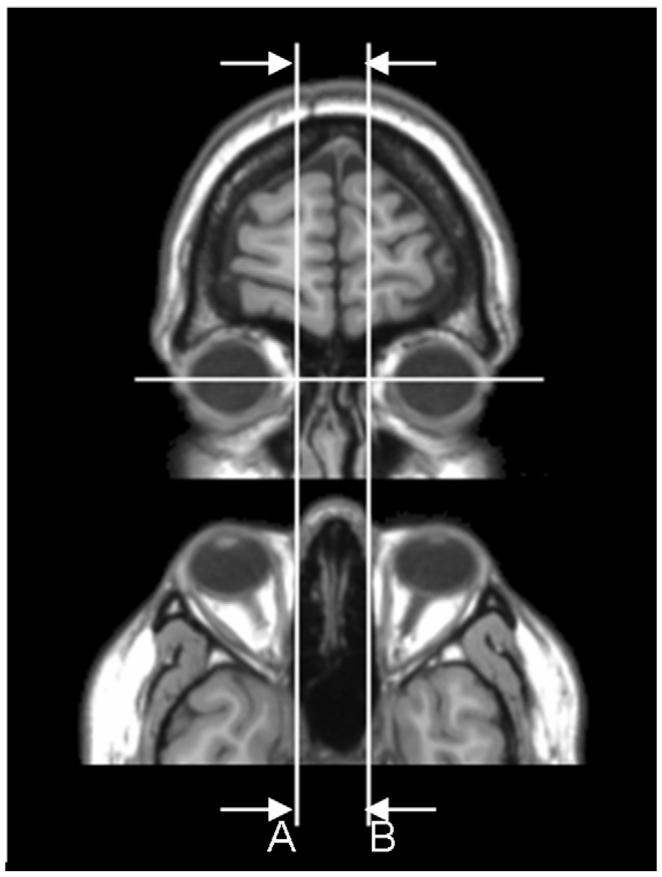
Measurement of inter-orbital distance in axial and coronal view. Top panel shows coronal view and lower panel shows the axial view. Measurements were made on the coronal view between A and B, with the axial view used for additional reference.

The MRIcro viewer software (http://www.cabiatl.com/mricro/) was used for MPA manual measurements. The axial slice which displayed the largest diameter for both eyes was selected for measurements of the distance between the orbits. The inter-orbital distance was defined as the most medial point of the orbits located at the point of intersection between nasal bone and eyeball [14], [16], see Figure 1. In this way, the inter-orbital distance was taken as the medial inter-ocular distance, since MRI scan taken at this position have the largest eyeball diameter. We conducted all measurements “blind” to diagnosis and performed an analysis of covariance in SPSS with T1 protocol as covariate to compare groups on optical parameters. We also performed independent t-tests to quantify group differences on optical parameters of MPA between children with autism and controls. Intra-rater reliability measurements were performed on 10 scans repeated after 1 week.

### Voxel-wise analysis

Images were preprocessed using SPM2 software. The preprocessing follows the optimized method [66] except that Expecta]?>tion Maximization Segmentation (EMS) [67], [68], [69] was used for multi-spectral segmentation of the PD/T2 data (http://www.medicalimagecomputing.com/downloads/ems.php). For this method, the PD/T2 images were segmented into tissue maps representing grey matter, white matter and CSF and linearly normalized to standard space to create customized study-specific templates. Next, as our study examined grey matter only, the grey matter maps in raw space were normalized non-linearly to the study-specific template. Finally, the normalized and segmented PD/T2 images were modulated with their Jacobian determinants to reflect their original volumes in native space following Good's optimized method [66]. These modulated images were used in the subsequent statistical analysis.

We conducted voxel-wise linear regression of grey matter volume and inter-orbital distance in each group separately using Cambridge Brain Activation software (CAMBA). Modulated grey matter maps from the preprocessing step were used to assess the significance of three-dimensional cluster statistics by non-parametric permutation testing [70]. The non-parametric linear modeling method used does not make any assumption on the smoothness of data. Therefore the data were not smoothed before the statistical analysis. The statistical thresholds were corrected for multiple comparisons by controlling the ‘family wise error rate’ and results accepted when the number of false positive clusters (FPC) expected under the null-hypothesis was <1. Total brain volume and age were entered as covariates. The clusters derived from these analyses were used to define volumes-of-interest to be extracted from both autism and control groups for confirmatory correlation analyses and to test for any group difference in regional volume.

### Volume-of-Interest extraction

Regions found to correlate significantly with inter-orbital distance in each group were exported as separate masks. Each mask was applied to the modulated grey matter maps of both control and autism group, and individual grey matter volume within this volume-of-interest were extracted.

## Results

**Figure 2:**
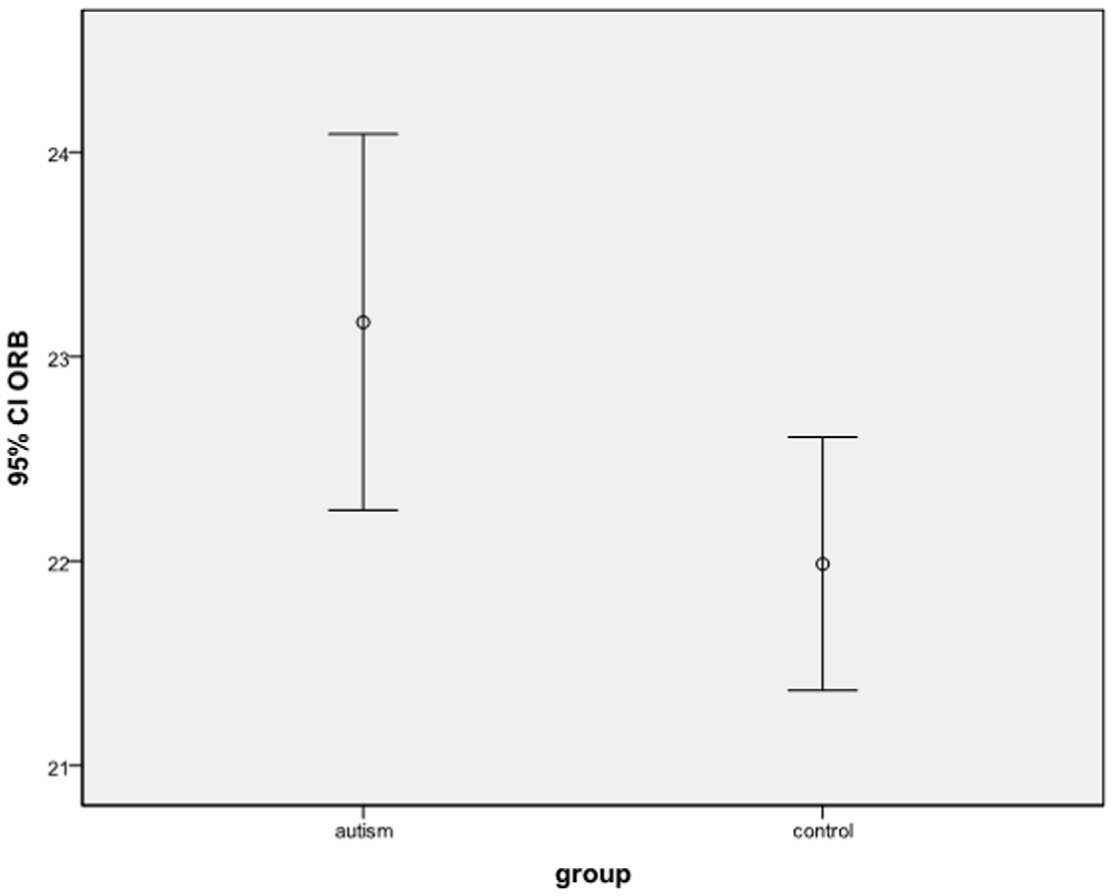
Group differences in inter-orbital distance (n = 91). CI, 95% confidence interval; ORB, interorbital distance in mm.

Inter-class coefficient ICC for MPA measurements performed 1 week apart was 0.95. There was no significant group difference in age, verbal IQ or total brain volume. As shown in Table 1 and Figure 2, the autism group had significantly greater inter-orbital distance than typically developing controls. This result was independent of the sequence used to acquire T1 data. In addition inter-orbit distance was independent of symptom severity, as scores on each domain of the ADI-R © WPS showed no significant correlation with inter-orbital distance. Excluding 6 children who did not reach ADI-R algorithm cut-off for autism did not alter the results.

**Table 1:** Sample characteristics (n = 91).

	Group	N	Mean	Standard Deviation	t-test
Age (years)	Autism	36	11.4	2.7	t = 1.278
	Control	55	10.7	2.7	p = 0.205
Verbal IQ	Autism	36	112	17.7	t = −1.227
	Control	51	117	18.1	p = 0.222
Brain volume (ml)	Autism	36	1450	105	t = 0.405
	Control	55	1440	108	p = 0.687
Inter-orbital distance (mm)	Autism	36	23.2	2.71	t = 2.156
	Control	55	22.0	2.29	p = 0.035

### Voxel-wise linear regression

**Figure 3:**
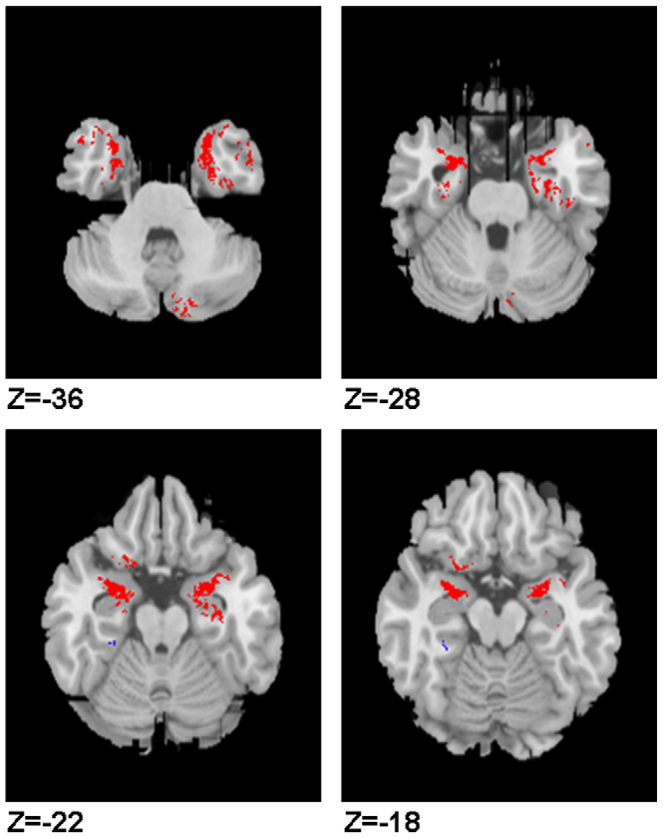
Voxel-wise correlates of inter-orbital distance in autism. Axial view of grey matter brain images. Left of the panel is left hemisphere. Red highlights clusters where inter-orbital distance is positively correlated brain volume in the autism group. Z co-ordinate is given in MNI space.

**Figure 4:**
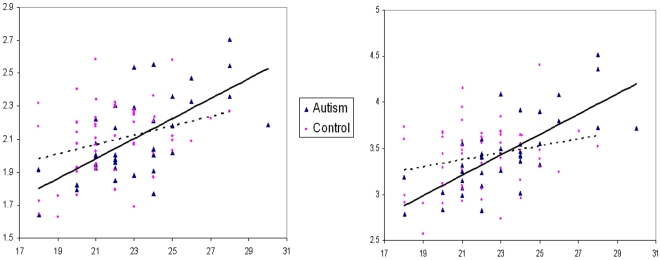
Scatter plot of grey matter volume against inter-orbital distance of a cluster in left and right amygdala region. Inter-orbital distance in mm. Grey matter volume in ml. Autism group represented by triangles and controls by circles. Left amygdala cluster volume correlation with inter-orbital distance is shown in the left panel. Right amygdala cluster correlation is shown in the right panel.

For the autism group only, in a voxel-wise linear regression analysis we found a significant positive correlation between inter-orbital distance and the grey matter volume of the bilateral amygdala, extending to unci and inferio-medial poles of the bilateral temporal lobes and left orbito-frontal lobe (p<0.001), see Figure 3. These findings held following confirmatory analysis of Pearson correlation co-efficients in SPSS using extracted volume-of-interest: left orbito-frontal r = 0.61, p<0.001; left amygdala r = 0.65 p<0.001; right amygdala r = 0.73, p<0.001, see Figure 4. Voxel-wise analysis also suggested a negative correlation between inter-orbital distance and a small cluster in the bilateral basal ganglia and posterior midline cortices in the autism group, but these did not survive volume-of-interest confirmatory analysis. No positive correlation in voxel-wise correlation was demonstrable in the control group at this level of significance, although volume-of-interest testing in the control group did suggest a weak positive correlation between inter-orbital distance and left amygdala (r = 0.295 p<0.05) and orbital frontal lobe (r = 0.267, p<0.05), see Figure 4. Moreover, when these co-efficients were converted to Z scores the correlations in the autism group were confirmed to be significantly greater than the control group: left amygdala z = 2.12, p = 0.03 (2-tailed); left orbito-frontal z = 1.96, p = 0.05 (2-tailed). Voxel-wise analysis indicated a negative correlation between inter-orbital distance and a cluster in left cerebellum of control group, however this did not survive confirmatory volume of interest analysis. Independent t-tests indicated no significant group difference in any volume-of interest including the amygdala based clusters, t>−3.5, p = 0.7 for both comparisons, see Table 2. Excluding the 6 children who did not reach ADI-R algorithm cut-off for autism did not alter the results. See Table 2, Figures 3 and 4.

**Table 2:** Brain correlates of inter-orbital distance.

Region		MNI Co-ords		Volume (ml)	
	x	y	z	Autism	Control
Right Amygdala	30.7	−1.8	−30.3	3.4±0.4	3.4±0.4
Left Amygdala	−26.7	−2.2	−29.0	2.1±0.3	2.1±0.2
Left orbito-frontal	−22.6	9.5	−16.2	0.30±0.03	0.30±0.03

## Discussion

The major finding in our study is that children with autism spectrum had significantly larger inter-orbital distance than the typically developing control group. In the autism group, inter-orbital distance was significantly positively correlated with the volume of the bilateral amygdalae, extending to infero-medial temporal lobes and orbito-frontal lobe. These results, covarying for age and total intracranial volume were confirmed in a volume-of-interest correlation analysis in SPSS. The increase in inter-orbital distance in the autism group was independent of symptom ‘scores’ on the diagnostic algorithm of the ADI-R.

Cohen has written extensively on malformations of the craniofacial regions [18] including how the optical system matures during brain growth. He noted that at birth, “on the one hand, the distance between the orbits increases; on the other hand, the optic angle decreases from 71°to 68°” in adulthood. Thus, if we refer to this trajectory of optical development, we speculate that the increased inter-orbital distance in the autism group is compatible with earlier head size expansion during infancy. This is because as the neurocranium enlarges, there is antero-posterior expansion of the cranial base, together with growth of the spheno-ethmoidal and spheno-frontal sutures such that the inter-orbital distance increases by as much as 50% of adult size by age 3 [18], [20].

The dominant direction of cranial base expansion is antero-posterior [18] and evidence is that children with autism have greater early expansion leading to larger head circumference in early childhood [21], [22], [23] is compatible with our data. Thus, although by late childhood global brain volumes are not appreciably different in our group of children with autism compared to typically developing peers [24], [25], this current phenomenon may signify dysplastic and/or disproportional growth in infancy, a critical period when early language, motor and social milestones are normally attained.

In the autism group, grey matter volume of the amygdalae (extending bilaterally to its ventro-medial fusion to the head of hippocampus (unci) and inferior pole of superior temporal lobes) was positively correlated with inter-orbital distance. This was not observed in the typical control group. The inference is that in this group, the growth of midline bony and brain regions are tightly linked; that is, regions involved in the regulation of socialization, emotion and memory [26], [27], [28], [29] appear to enlarge with the visual system. The uncus is the site of fusion of hippocampal head and ventro-medial amygdala and if lesioned in Rhesus monkeys leads to social indifference, lack of appropriate aggression and submissiveness, and eventually even expulsion from the colony [30]. Both uncus and amygdala are activated when socially phobic individuals look at angry or contemptuous faces [31], and this response extends to hippocampus and temporal pole during anticipatory anxiety [32]. This ethological evidence squares with a putative role of the amygdala in the pathenogenesis of autism [33], [34], [35], [36] (Baron-Cohen et al., 2000, Shaw et al., 2005). Brothers originally proposed a “social brain” network after observing that social intelligence was linked to the amygdala, the orbito-frontal cortex and the superior temporal gyrus [29]. More recent studies have observed an enlargement of the amygdala in toddlers who are eventually diagnosed with autism [37], [38]. However studies of amygdala size in older children and adults with autism have been much more inconsistent, finding no difference [36] or even smaller amygdala relative to controls [39], [40]. This has lead to the hypothesis that in early post-natal development of children with autism, the amygdala increases in size but more typical age-related expansion found in unaffected children does not occur [38]. Our results are consistent with this idea.

We previously examined regional brain volumes in these children and did not identify a between-group difference in amygdala volume in the autism group compared to typically developing controls [25]. In the present analysis, again we found no evidence for a group difference in volumes extracted from the amygdala region correlated with inter-orbital distance. However small in absolute terms, the increase in inter-orbital distance linked to greater amygdala volumes identified here suggests a record of early brain dysmaturation involving the amygdala. For typically developing controls no such strong relationship emerged. Could early amygdala enlargement bear ill portent? Resonating with this idea is very recent evidence showing an inverse relationship between right amydala volume of infants scanned at 6 months and receptive and expressive language ability at age 2, 3 and 4 years of age [41].

We capitalized on voxel-based morphometry to explore the relationship between brain structure and MPAs for each individual. Thus far, just one other study has used MRI to examine the relationship between brain morphology and MPAs and they performed this in individuals diagnosed with schizophrenia [15]. Dean and colleagues examined a first-episode schizophrenia epidemiological cohort and reported total MPA score to be negatively correlated with prefrontal cortical volume but positively correlated with basal ganglia/thalamic volume. Based on the total MPA score rather than on discrete cranio-facial parameters, they interpreted their findings as indicating cranial dysmorphogenesis [42] since first trimesteric/early second trimesteric aberrant development is regarded as part and parcel of the schizophrenia diathesis. Because the embryonic notochord is a midline structure, midline cranio-facial development may also be compromised should there be aberrant brain development during fetal life. Although our data are not directly comparable, we speculate that the process governing amygdala and optical differentiation from neural crest cells is tightly orchestrated, perhaps at no other time as vital as within the first 3 years of life [43].

In terms of possible casual mechanisms, convention holds that minor physical anomalies develop during the first trimester [8], [11]. Increased inter-orbital distance as in hypertelorism is associated with around 550 conditions with origins in prenatal life [44], including Coffin-Lowry syndrome, an X-linked mental retardation syndrome associated with autism [45]. Genetic abnormalities are certainly part and parcel of the etiology of these anomalies [3], [46] but hypertelorism is causally heterogeneous and many genetic pathways can go awry [47]. In addition environmental exposures, such as rubella, infection, anoxia, bleeding, fetal distress, dietary deficiency, and toxemia, have been implicated in MPAs [48], [49] [50]. In turn just such a range of prenatal environmental insults have been reported to increase the risk of autism in offspring such as case reports of prenatal infection [51], [52], [53], [54], [55], [56] and maternal stressors during gestation [57], [58], [59]. Thus the multiple genetic and environmental influences on early life development which can shift brain maturation towards an autistic trajectory appear to coincide with the pressures driving cranio-facial, including optical, dysmorphology.

We acknowledge inherent limitations of the methodology concerning the choice of reference axis for performing MPA measurements. We had considered a range of choices for baseline axis, all of which leverage on reliable and easy identification by external inspection or from bony X-rays. For example the orbito-canthal plane is formed by joining the external angle of the eye (canthus) to the ear canal (external auditory meatus). Alternatively, the Frankfurt horizontal plane according to the Boas atlas is classically taken as including the base of the orbits and the external auditory meatus and parallel to the horizontal plane, a plane which is commonly adopted by anthropologists in archaeological excavations [60] as well as by paediatric oto-laryngological and maxillo-facial surgeons today [61]. Although our sample size was sizeable, it consisted of high-functioning subjects with autism spectrum who all had normal IQ, attended normal school, and originated from a community programme. This may limit the wider generalisability of the findings since the majority of persons with autism have below-normal IQ and these relatively able children may have come to attention because of the degree of symptomatology or other parent concerns. On the other hand, MPAs have been more usually associated with congenital or genetic syndromes, and to our best knowledge these high functioning children involved in our study did not carry such diagnoses. However formal testing would be important to carry out in future studies.

In summary we found significantly increased inter-orbital distance in children with autism which was highly correlated with larger amygdala size in intellectually able children with autism. Thus MPAs may indicate early disruption of brain development even in intellectually able. We note that other non-brain dysmorphologies merit evaluation too and are in the process of extending our range of measurements. Replication is awaited in other study populations but these novel findings suggest MPAs may in time provide a useful additional biomarker for autism.
